# Recent Advances in Lipopolysaccharide Recognition Systems

**DOI:** 10.3390/ijms21020379

**Published:** 2020-01-07

**Authors:** Lalita Mazgaeen, Prajwal Gurung

**Affiliations:** 1 Inflammation Program, University of Iowa, Iowa City, IA 52242, USA; lalita-mazgaeen@uiowa.edu; 2 Department of Internal Medicine, University of Iowa, Iowa City, IA 52242, USA; 3 Interdisciplinary Graduate Program in Human Toxicology, University of Iowa, Iowa City, IA 52242, USA; 4 Immunology Graduate Program, University of Iowa, Iowa City, IA 52242, USA

**Keywords:** LPS, endotoxin, TLR4, TRPA1, TRPV4, caspase-11

## Abstract

Lipopolysaccharide (LPS), commonly known as endotoxin, is ubiquitous and the most-studied pathogen-associated molecular pattern. A component of Gram-negative bacteria, extracellular LPS is sensed by our immune system via the toll-like receptor (TLR)-4. Given that TLR4 is membrane bound, it recognizes LPS in the extracellular milieu or within endosomes. Whether additional sensors, if any, play a role in LPS recognition within the cytoplasm remained unknown until recently. The last decade has seen an unprecedented unfolding of TLR4-independent LPS sensing pathways. First, transient receptor potential (TRP) channels have been identified as non-TLR membrane-bound sensors of LPS and, second, caspase-4/5 (and caspase-11 in mice) have been established as the cytoplasmic sensors for LPS. Here in this review, we detail the brief history of LPS discovery, followed by the discovery of TLR4, TRP as the membrane-bound sensor, and our current understanding of caspase-4/5/11 as cytoplasmic sensors.

## 1. Introduction

**Figure 1:**
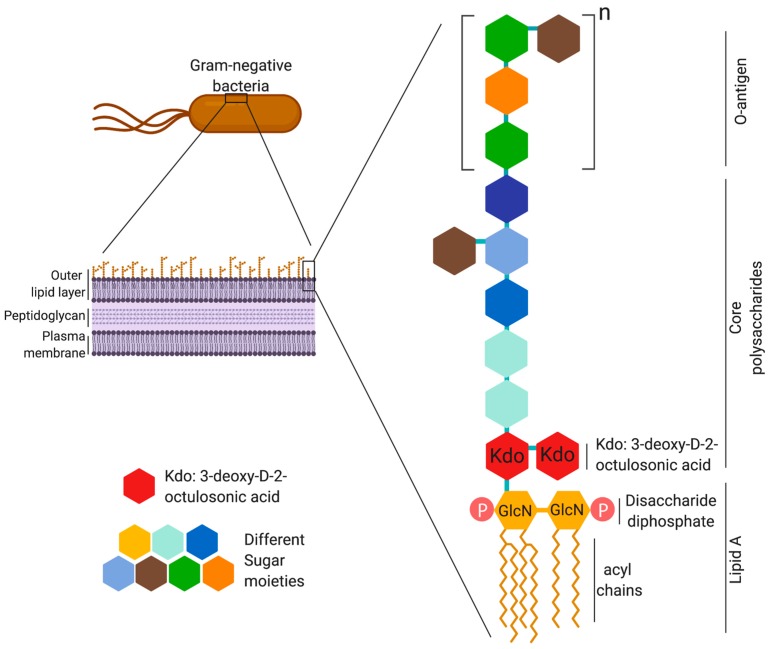
Structural details of lipopolysaccharide from a Gram-negative bacterium. Lipopolysaccharide (LPS) provides structural and functional integrity to outer membrane of Gram-negative bacteria. LPS is an amphipathic molecule with a general structure consisting of three different regions: hydrophobic lipid A, core polysaccharide, and O-antigen (repeats of polysaccharide chain, where n can be up to 40 repeats). Lipid A consist of bisphosphorylated diglucosamine backbone substituted with six acyl chains that are attached by ester or amide linkage.

Lipopolysaccharide (LPS) is an important cell wall constituent of Gram-negative bacteria and is vital for bacterial cell integrity, viability, and defense against environmental stress [1]. Lipopolysaccharide is highly conserved among almost all Gram-negative bacteria and is a potent inducer of inflammatory responses. It exists ubiquitously in the environment and, thus, LPS can directly modulate the immune system and susceptibility to disease [2]. Lipopolysaccharide is a heat stable amphiphilic molecule composed of three regions: lipophilic lipid A, hydrophilic polysaccharides or oligosaccharide core, and O-antigen (Figure 1). The lipid A portion of LPS has been demonstrated to be the immunostimulatory moiety of LPS. Studies of various Gram-negative bacteria show that the structure of lipid A is diverse among bacterial species, and the number of acyl chains (fatty acid) determines the immunostimulatory capacity of LPS. Nonetheless, highly immunostimulatory LPS comprises comparatively similar lipid A with six acyl chains. For example, both *Escherichia coli* and *Salmonella enterica* serovar Typhimurium comprise hexa-acylated lipid A which are highly immunostimulatory [3,4,5].

The discovery of LPS dates back to the eighteenth century with the search for a substance found in putrid matter that was believed to cause fever. Later, Robert Koch (1843–1910) showed that various diseases were caused by bacteria. Richard Friedrich Johannes Pfeiffer (1858–1910) demonstrated that some bacteria consisted of a heat stable, non-volatile pyrogenic substance that caused disease and termed it “endotoxin” to distinguish it from exotoxins that are released by bacteria. Subsequently, endotoxin was shown to characterize Gram-negative bacteria. By the 1940s, pure extracts of endotoxin were prepared and demonstrated to be made of a small portion of lipid A and polysaccharide, hence named lipopolysaccharide. In the 1980s, Tetsuo Shiba et al. [6,7] synthesized free lipid A molecule and proved it to be the endotoxic center of LPS.

Intensive research in the field of innate immunity has led to the identification of a myriad of pattern recognition receptors (PRRs) on host immune cells that recognize non-self-molecules, i.e., pathogen-associated molecular patterns (PAMPs) derived from various pathogens including LPS. Extracellular LPS is a potent PAMP recognized by toll-like receptor-4 (TLR4) which is a PRR present on the surface of phagocytic cells like macrophages, neutrophils, and dendritic cells. Recognition of LPS by TLR4 induces a signaling cascade that eventually induces inflammation and production of the pro-inflammatory cytokines that help eliminate invading pathogens [8,9]. Conversely, excessive production of pro-inflammatory cytokines leads to life-threatening pathological consequences such as septic shock [10,11].

While TLR4 was thought to be the only sensor for LPS, recent studies have provided insight into two TLR4-independent LPS recognition systems: transient receptor potential (TRP) channel-dependent sensing of extracellular LPS and caspase-4/5/11-dependent sensing of intracellular LPS. Extracellular LPS sensed by TRP channels present on the neuronal cells drives neurogenic inflammation and pain in mice [12]. Caspase-4/5 in humans and caspase-11 in mice sense intracellular LPS within the cytoplasm of innate immune cells, such as macrophages, to drive production of pro-inflammatory cytokines IL-1β and IL-18 and inflammatory cell death, termed pyroptosis [13,14,15].

This review provides an overview of the current state of knowledge regarding LPS structure and immunogenicity. We further discuss the literature and provide specific details about the TLR4-dependent and TLR4-indepdent LPS recognition systems that have been uncovered recently.

## 2. LPS Structure and Immunogenicity

Studies of various Gram-negative bacteria suggest a common general structure of LPS. The membrane-embedded lipophilic lipid A is usually composed of a dimer of glucosamine (D-GlcN) attached to acyl chains by ester or amide linkages. Lipid A is covalently attached to hydrophilic anionic groups, 3-deoxy-d-manno-2-octulosonic acid (Kdo) in the core region together with L-glycero-D-manno-heptose (l,d-Hep) and hexoses and hexosamines. In most Gram-negative strains, the core region is attached to the repeated units of saccharides called O-polysaccharides or O-antigens [1,16,17]. O-antigens vary among bacterial strains and give bacteria a rough (R-type) or smooth (S-type) phenotype. O-antigens are truncated or lacking in R-type when compared to S-type Gram-negative bacteria. As the outermost part of LPS, O-antigens are responsible for bacteria evading the immune system, particularly the complement system of the host (e.g., *Salmonella enterica* serovar Typhimurium) [9,18]. Lipid A as well as the polysaccharide regions are able to induce potent immune responses [16,19,20,21,22]. The lipid A component, independent of the polysaccharide portion, is responsible for various pathophysiological effects including toxicity, mitogenicity, complement reactivity [23,24], and Limulus lysate gelation [7,25,26]. Galanos et al. [25] demonstrated that solubilized lipid A administered to mice and rabbits induced toxicity and pyrogenicity. Furthermore, soluble lipid A demonstrated the properties of intact LPS, i.e., lethal toxicity and pyrogenicity induced by lipid A was comparable to their corresponding parent LPS. Furthermore, chemically synthesized *E. coli* lipid A has demonstrated identical endotoxic activities as natural free lipid A, further supporting lipid A as the endotoxic center of LPS [19,25,26].

The immunogenic potential of LPS varies among bacterial species and is mostly determined by the structure of lipid A [7,27]. Based on the potency of the pro-inflammatory reaction, LPS can be agonistic, weakly agonistic or antagonistic. Generally, the LPS present in major Gram-negative bacteria are agonistic and hexa-acylated (six acyl chains are esterified with disaccharide backbone) and are therefore potent immunostimulants. The structure of Lipid A can vary in number, length, position of esterified acyl chain, and number of phosphate groups, resulting in various degrees of immunogenicity [3,28]. The tetra-acylated lipid A structure found in *Yersinia pestis* grown at 37 °C has very low immunogenic activity and has been shown to be a TLR4 antagonist [29]. Similarly, *Francisella tularensis* expresses lpxD1 lipid A biosynthase acyl transferase at 37 °C which synthesizes the tetra-acylated lipid A of a longer acyl chain length with lower activity [30].

Studies have demonstrated that, in addition to lipid A, charged molecules, such as phosphate groups, have a direct impact on the interaction between phagocytic cells and cytokine production. For example, both hexa-acyl groups and two phosphate groups of *E. coli* are required for full activation of the TLR4/MD2 complex [19]. Similarly, *Francisella tularensis* demonstrates weak endotoxic activity when one or both phosphate groups are lacking, compared to hexa-acylated *Escherichia coli* lipid A with two phosphate groups at the 1 and 4 positions [31,32]. In addition, several studies have demonstrated the contribution of inner core sugars (two Kdo moieties) of LPS in inflammatory responses [22]. Studies with *N. meningitids* LPS variants and the synthetic lipid A variants showed the important role of two Kdo with lipid A; in fact, the response of human monocyte-derived dendritic cells to *N. meningitids* Re-LPS was higher compared to lipid A alone [33]. Similarly, the production of proinflammatory cytokines, like TNFα and IFN-β, were significantly higher in the murine macrophages when stimulated with synthetic *N. meningitids* Re-LPS compared to lipid A, and similar results were observed with *E. coli* [34] and *Salmonella* LPS [35]. Thus, the structural features of these components are directly related to the virulence of Gram-negative bacteria and their ability to evade immune detection. Understanding the intricate process of LPS recognition by the host immune system can provide important insight into the nature and magnitude of inflammatory responses.

## 3. LPS Recognition by TLR4

The critical role of the innate immune system is to detect and recognize the presence of foreign invaders and promote phagocytic killing of microorganisms. The primary targets of LPS are the phagocytic cells of the immune system including tissue macrophages, peripheral monocytes, neutrophils, and dendritic cells. Lipopolysaccharide activates macrophages and induces the production of several pro-inflammatory cytokines, some of which are responsible for inflammation-induced fever. Although controlled production of these inflammatory mediators is required for clearance of invading pathogens, uncontrolled production of inflammatory cytokines can provoke fatal consequences such as septic shock [9,36]. Therefore, fine tuning of pro- and anti-inflammatory mediators is required for proper immune function and homeostasis maintenance. In normal physiological conditions, several molecules, including sCD14 [37], lysozyme [38], BPI protein [39], collectins [30], lactoferrin [40], tissue factor pathway inhibitors [41], and cationic antimicrobial protein CAP18 [42], actively regulate low levels of LPS and remove it from circulation to limit inflammatory responses [43,44,45].

**Figure 2:**
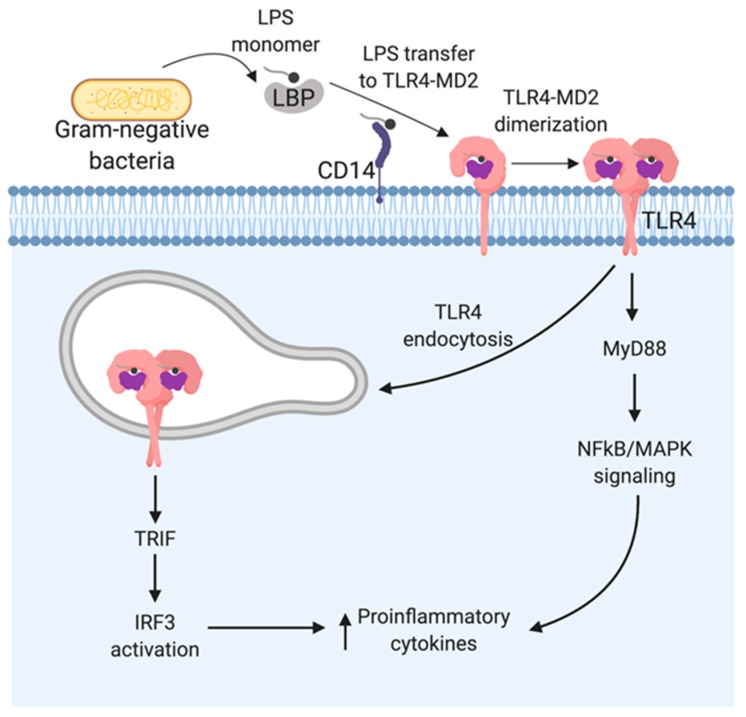
LPS recognition by toll-like receptor 4 (TLR4). Serum protein LBP (LPS-binding protein) binds monomer of LPS from Gram-negative bacteria and delivers it to a CD14 molecule that can be either soluble or membrane-bound (glycosylphosphatidylinositol-anchored protein). CD14 transfers LPS to the ectodomain of the TLR4/MD-2 receptor complex which leads to homodimerization of TLR4. This change in conformation leads to dimerization of the cytoplasmic TIR-domain (Toll-interleukin-1 receptor) that provides a binding site for MyD88. This activates the transcription factor nuclear factor-κB (NF-κB) and MAPK (mitogen-activated protein kinase) and transcription of various proinflammatory cytokines. In addition, the endocytosis of the LPS-TLR4/MD-2 complex leads to the TRIF-dependent signaling pathway that mediates the induction of interferon regulatory factor 3 (IRF3) and type-1 interferons.

TLR4, present on the surface of various phagocytic cells, is the specialized cellular sensor for extracellular LPS (Figure 2). Efficient LPS recognition and production of inflammatory mediators by TLR4 requires an orchestrated action of various accessory proteins such as LPS-binding protein (LBP), CD14, and MD-2 [46,47,48]. Although the exact nature of the interaction between LPS and TLR4/MD-2 is somewhat obscure (structure–activity relationship), various studies have been able to unravel the complex process to some extent. Initiation of LPS recognition begins with dissociation of the LPS monomer from the aggregates by LPS-binding protein (LBP) which traffics it to CD14 (glycosylphosphatidylinositol-anchored protein), present on most phagocytic cells (except dendritic cells) that make use of soluble CD14 (sCD14). CD14 then carries and loads LPS to the TLR4/MD-2 receptor complex.

Various studies have been conducted to understand the sequential molecular and structural basis of LPS recognition by TLR4 [49]. Lipopolysaccharide aggregates (i.e., micelles) are often secreted by Gram-negative bacteria in the form of outer membrane vesicles (OMVs) [50,51]. Extraction of LPS monomer from the LPS micelles requires concerted action of both LBP and CD14. LBP is a glycoprotein with elongated structure consisting of N-terminal and C-terminal domains connected by a central domain. Using negative-stain TEM imaging and mutagenesis experiments, Ryu et al. showed that the N-terminal domain of LBP binds to LPS aggregates with high affinity [52,53]. Mutations at the N-terminal conserved basic amino acids (Arg73, Lys67, Lys69) and basic amino acids (Arg119, Lys120, and Lys124) previously shown to facilitate LPS binding at N-terminal [54] abolished binding of LBP to LPS. In a reaction mixture of LPS micelles with LBP or CD14, only LBP molecules bound to the surface of thread-like LPS micelles, suggesting CD14 cannot directly interact with LPS aggregates. However, CD14 can bind to LBP-LPS aggregate and extract LPS monomers from the LBP–LPS aggregate. Interestingly, CD14 bound to the C-terminal end of LBP separated from the LBP N-terminal end bound to LPS micelles. Furthermore, using total internal reflection fluorescence (TIRF), Ryu et al. demonstrated the requirement of the physical interaction between C-terminal of LBP and CD14 for the transfer of LPS monomer to CD14 [53,55]. These experiments support the hypothesis that LPS monomers are somehow transported from the N-terminal end of LBP to the C-terminal end whereby CD14 can then extract these LPS monomers. CD14 carrying an LPS monomer then dissociates from LBP and subsequently facilitates LPS transfer to MD2/TLR4 complex. When TLR4–MD2 complex or MD2 alone was introduced to surface immobilized CD14 complexed with Cy5 labelled LPS, Cy5 fluorescence intensity drastically decreased only with the addition of TLR4–MD2, suggesting the requirement of the TLR4–MD2 complex for LPS extraction from the CD14–LPS complex. Furthermore, LPS binds with TLR4–MD2 with higher affinity compared to MD2 alone [53,56]. Thus, LPS transfer to the TLR4–MD2 complex is a one-step process in that MD2 alone cannot receive/extract LPS from CD14 [53]. Park et al. [57] demonstrated that LPS binding leads to the dimerization of the TLR4/MD2–LPS complex, and LPS interacts with the hydrophobic pocket in MD-2 where five of six lipid chains are buried deep inside the pocket and one is exposed to the surface which interacts with a second TLR4 molecule. This leads to further structural changes in MD-2 that supports the dimerization of the TLR4–MD-2 complex [57,58]. Binding of LPS to TLR4–MD2 depends on the structure of LPS as LPS from *R. sphaeroides* does not lead to dimerization of TLR4 compared to LPS from *E. coli* [59]. Recently, Hubert el al. [60] developed a computational model to study the thermodynamics and affinity of the receptor complex in the process of LPS transfer to TLR4 and suggested that the lipid affinity increases along the TLR4 signaling cascade. The affinity for lipid A binding to CD14 (136 ± 13 kJ mol^−1^) is less than the affinity for MD2 (156 ± 11 kJ mol^−1^) and lipid A binding to the MD2/TLR4 complex (~400 kJ mol^−1^) is the most thermodynamically favored event [61]. Furthermore, Phe126 residue in MD2 is important for the transfer of LPS from CD14 to the TLR4–MD2 complex which acts as a hydrophobic switch to mediate the signaling at the TLR4 complex dimerization interface [60,61].

TLR4–LPS binding promotes homodimerization of the ectodomains of TLR4, and the subsequent structural and conformational changes induce dimerization of cytoplasmic TIR (Toll-interleukin-1 receptor) domains. The dimerized TIR domain structure is recognized by the downstream adaptor proteins MyD88 (myeloid differentiation primary response gene 88) and TIRAP (TIR domain-containing adaptor protein), leading to the formation of a protein complex, Myddosome, along with several other serine-threonine kinases of the IRAK family [62]. Myddosome mediates the signaling cascade that leads to the activation and subsequent translocation of transcription factor NF-κB and expression of pro-inflammatory cytokines such as TNF-α, IL-1β, and IL-6 [63,64]. Furthermore, the MyD88-dependent pathway also activates the downstream MAP kinase pathway that leads to AP-1 transcription and expression of other pro-inflammatory cytokines [65,66].

In addition to signaling at the cell surface, TLR4 signaling within the endosomes begins with the assembly of TRIF (TIR domain-containing adaptor inducing IFN-β) and TRAM (TRIF-related adaptor molecule), leading to the assembly of triffosome which promotes activation of IRF3 (interferon regulatory factor 3) and induction of type-1 interferons [66,67,68]. Thus, signaling cascades downstream of TLR4 in distinct subcellular sites leads to the production of different pro-inflammatory cytokines.

Sugar-dependent receptors such as C-type lectins on macrophages and dendritic cells play important roles in innate immunity. They recognize microbial polysaccharides and enhance signaling through TLRs [69]; for instance, *Neisseria gonorrhea* lipooligosaccharide (lacks O-antigen) lipid A is detected by the TLR4/MD2 complex, and the oligosaccharide (phenotype C) is detected by sugar-dependent receptor C-type lectin DC-SIGN on dendritic cells (in human). Neisseria has several variants that differ in the terminal carbohydrate moieties of their lipooligosaccharide which play a vital role in immunomodulatory properties. These variants induce similar DC maturation, but the differences are seen in the cytokine profile and subsequent T-cell polarization depending on the differential C-type lectin usage [70]. C-type lectin, SIGNR1 in macrophages of mice, enhances TLR4 oligomerization when macrophages are stimulated with *E. coli* or *S. typhimurium* [69,71]. Using Ba/F3 cells expressing SIGNR1 and LPS from rough mutants of *S. typhimurium,* Nagaoka et al. showed that the SIGNR1-recognized polysaccharide part of LPS enhanced TLR4–MD2 complex oligomerization and subsequent production of proinflammatory cytokines [69]. Similarly, Saunders et al. demonstrated that mice deficient in SIGNR1 were resistant to LPS shock and DSS-induced colitis with reduced colon damage and production of proinflammatory cytokine [72]. *Signr1*-/- mice are partially protected from LPS-induced mortality; however, both *Tlr4*-/- and *Tlr4*-/- × *Signr1*-/- DKO mice are equally resistant to LPS shock [72]. Thus, it is not clear whether LPS-induced SIGNR1 signaling can occur independently of TLR4, and additional comparative studies should address this in the future.

For decades, TLR4 remained the sole immune receptor for LPS, and the importance of TLR4 in pathogen recognition and homeostasis maintenance have been studied extensively. TLR4-deficient mice are highly susceptible to different strains of Gram-negative bacteria because of the inability to recognize and initiate a proper immune response [73]. Conversely, TLR4-deficient mice are highly resistant to LPS shock [74]. These studies highlight the importance of TLR4 in LPS recognition. Recent advances have identified TLR4-independent LPS recognition systems that include the transient receptor potential (TRP) channels and caspase-4/5/11 sensors.

## 4. LPS Recognition by TRP Channels

**Figure 3:**
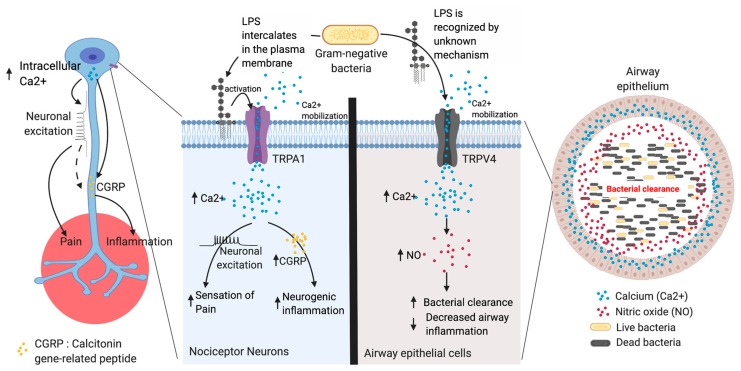
LPS recognition by transient receptor potential (TRP) channels. Transient receptor potential (TRP) cation channels are novel sensors of LPS that are present in the sensory neurons and epithelial cells. TRP channels can recognize LPS before initiation of TLR4 signaling during inflammation. TRPA1 in nociceptive neuron and TRPV4 in non-neuronal cells, such as airway epithelium in mice, are the most studied. LPS recognition by these TRP cation channels leads to an influx of Ca^2+^ ions that generate action potential firing (neuronal excitation) which activates intracellular signaling cascade and the release of signaling peptides (CGRPs) that generate pain during inflammation. In the airway epithelium, LPS can be sensed by TRPV4 and the subsequent increase in Ca^2+^ influx leads to the production of nitric oxide (NO) which facilitates the pathogen clearance from the airways.

Transient receptor potential (TRP) cation channels present in sensory neurons and epithelial cells have been demonstrated as novel LPS sensors that are sensitized and activated before the initiation of immune responses through TLR4 (Figure 3).

Transient receptor potential (TRP) channels are a large superfamily of ion channel proteins. They are divided into two groups and seven subfamilies according to the amino acid sequence homology [75]. Group 1 consists of five subfamilies: TRPC (canonical), TRPV (vanilloid), TRPM (melastatin), TRPA (ankyrin), and TRPN (NOMPC-like). Group 2 consists of two subfamilies: TRPP (polycystin) and TRPML (mucolipin). Transient receptor potential channels share common structural features composed of homologous tetramers, each with intracellular carboxy (C–) termini, six transmembrane (TM) domains, and an amino (N–) termini with regulatory motifs. Transient receptor potential ion channels are expressed in a wide variety of cells and are activated by diverse stimuli. They are cellular sensors of various environmental and intracellular stimuli [73] and play a critical role in cellular homeostasis. Most TRP channels are non-selective cation channels, and their activation leads to depolarization of resting membrane potential and activation of voltage-dependent ion channels which, in turn, change intracellular Ca^2+^ concentration. Recognition of pathogenic bacteria by these TRP cation channels in nociceptive neurons results in Ca^2+^ influx and action potential firing, leading to the intracellular signaling cascade responsible for release of signaling peptides that generate pain during inflammation [76,77]. The role of TRPA1 and TRPV1 in neurogenic inflammation is the most extensively studied and well established [78].

Gram-negative bacterial infections leading to inflammation are accompanied by somatic or visceral pain which is attributed to activation of nociceptors by inflammatory mediators released from immune cells and, until recently, was thought to be secondary to TLR-dependent immune activation [74,79]. However, various studies have shown that the neuronal activity in response to LPS occurs rapidly before the initiation of a pro-inflammatory immune response [78]. Meseguer et al. showed that the neurogenic inflammation and pain produced by LPS was dependent on the activation of functional TRPA1 channels and was independent of TLR4 activation [12]. Lipopolysaccharide activated the sensory neurons (as demonstrated by intracellular calcium measurement) isolated from the nodose and trigeminal ganglia of WT mice which was not affected by TLR4 deficiency [12]. Importantly, LPS-induced calcium influx and activation of these neurons were dependent on TRPA1 channels as demonstrated by lack of LPS-induced calcium influx in *Trpa1*-/- mice. Lipopolysaccharide-induced mobilization of calcium in the neurons triggered neuronal excitation and sensation of pain [12]. Calcitonin-gene-related peptide (CGRP, a neurogenic peptide) is produced in response to several neuronal stimuli and promotes neurogenic inflammation [12,80,81]. Lipopolysaccharide induced the production of CGRP in a TRPA1-dependent and TLR4-independent manner [12]. To address whether Ca2+ directly promotes CGRP production or neuronal excitation are precursors to CGRP will require further investigation [12]. TRPA1-mediated sensing of LPS is an evolutionarily conserved mechanism, as a role for TRPA1 in LPS sensing has been observed in *Drosophila melanogaster* [82]. Saldano et al. demonstrated that TRPA1 functions as a chemosensor of pathogenic cues and is responsible for aversive responses and gustatory-mediated avoidance to LPS in *Drosophila melanogaster* [83]. TRPA1 activation by LPS in neuronal cells was completely inhibited after polymyxin B treatment (a cationic cyclic antibiotic that binds to lipid A), suggesting an important role for the lipid A moiety in this process. The efficiency of TRPA1 activation and the inflammatory outcomes have been shown to vary according to the lipid A structure of LPS, i.e., LPS with asymmetrical hexa-acyl lipid A induced the strongest activation of TRPA1 as measured by calcium influx [12]. Furthermore, LPS treatment-induced TRPA1 currents in isolated membrane patches suggest a role for TRPA1 in sensing membrane perturbations [12]. These observations have been strengthened by a recent study demonstrating that LPS is able to be inserted into the cell membrane of TRPA1-expressing CHO cells [84]. Importantly, when LPS was added to lipid vesicles made from 1,2-dipalmitoyl-sn-glycero-3-phosphocholine (DPPC), decreased permeability of the vesicles was noted as measured by the fluorescent probe Laurdan (6-lauroyl,1-2-dimethylamino naphthalene) with an emission shift from a 400–525 nm wavelength. Thus, these studies directly support the hypothesis that LPS intercalates into cell membranes and promotes mechanical perturbations to activate TRPA1.

In addition to TRPA1, Boonen et al. demonstrated that LPS activates TRPV1 to promote an intracellular calcium influx in HEK293T cells transfected with recombinant human TRPV1 [85]. In the mouse dorsal root ganglion (DRG) neuron, the LPS response that was only partially blunted in *Trpa1*-/- neurons was completely abrogated in *Trpa1*-/-*Trpv1*-/- DKO neurons. This suggests involvement of TRPV1 in sensing LPS and neurogenic inflammation [85]. Moreover, using recombinant TRP expression in HEK293T cells, Alenmyr and colleagues demonstrated that, in addition to TRPA1 and TRPV1, LPS weakly activated TRPM3 and TRPM8 to promote calcium influx [86]. It should be noted that other studies have demonstrated expression of TLR4 in a subclass of TRPV1 containing trigeminal nociceptors and that LPS sensitized TRPV1 via TLR4-mediated signaling [87,88]. Thus, more extensive studies are needed to determine whether TRPV1, TRPM3, and TRPM8 are directly activated by LPS-induced membrane disruption or require TLR4 for their activation.

In addition to sensory neuronal TRP, a recent study has shown the presence of a non-neuronal TRP channel that can detect LPS; specifically, the TRPV4 channel in airway epithelium in mice can detect LPS [89]. TRPV4 is expressed in both murine and human airway epithelial cells [86,90]. Alipzar et al. demonstrated that murine airway tracheobronchial epithelial cells (mTECs), when stimulated with LPS, induced calcium influx that was dependent on TRPV4 and independent of TLR4 [89]. LPS-induced activation of TRPV4 channels in mTEC led to nitric oxide (NO) production that was subsequent to calcium influx. Importantly, LPS-induced calcium influx and NO production was intact in mTEC from TLR4-deficient mice. TRPV4-mediated LPS sensing is functionally important, as WT mTEC-mediated killing of non-pathogenic *E. coli* in vitro was blunted in *Trpv4*-/- mTEC. In vivo, LPS aerosol exposure induced significantly stronger ventilator responses (as measured by Penh value) and increased neutrophil and monocyte infiltration in the lungs of *Trpv4*-deficient mice when compared to WT controls. Thus, TRPV4-mediated early responses to LPS may be important in curbing unwanted inflammation in the lungs.

More recently, a role for TRPV4 has also been shown in macrophages using siRNA knockdown of TRPV4 or via chemical inhibition using HC-067047 [91] or GSK2193874 [92] (inhibitors of TRPV4) [93]. With both approaches, macrophages in the absence of functional TRPV4 had an attenuated response to LPS as demonstrated by reduced TNF, IL-6, and NO production. Further studies are needed to clarify whether LPS-induced activation of TRPV4 in macrophages are also TLR4 independent.

Altogether, these studies provide solid evidence that TRP ion-channels, such as TRPA1 and TRPV4, can quickly detect the presence of LPS to initiate acute inflammatory responses and provide a robust defense against invading pathogens.

## 5. LPS Recognition by Caspase-11

**Figure 4:**
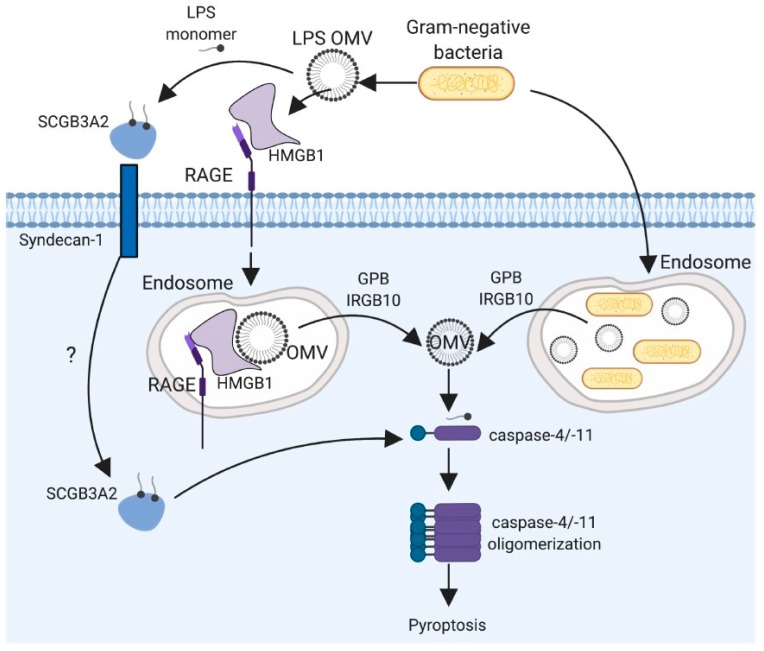
LPS recognition by caspase-4/-11. LPS can access cytosol when Gram-negative bacteria disrupt the phagolysosome or by the uptake of LPS-containing outer membrane vesicles (OMVs) released by live bacteria and their subsequent lysis. The uptake of LPS/OMV is facilitated by HMGB1. The HMGB1–LPS complexes are internalized into endosomes through a receptor for advanced glycation end-products (RAGEs). LPS is released into cytosol after HMGB1 permeabilizes the phospholipid bilayer under acidic conditions. In addition, host GBPs and IRGB10 can facilitate the release of LPS from endosomes. Secretoglobin (SCGB)3A2 binds LPS, and the SCGB3A2–LPS complex can access cytosol (via an unknown mechanism indicate by “?” in the figure) through binding with the cell surface receptor syndecan-1. Once the LPS is delivered into the cytosol, caspase-4/-11 senses LPS, leading to its oligomerization and induction of pyroptosis.

As discussed above, TRP channels and TLR4 can both recognize LPS independently to initiate non-redundant signaling pathways; however, both of these receptors are membrane bound and, as a result, recognize LPS that are either extracellular or within the endosomes. So, what happens to the LPS that may find its way into the cytoplasm of the cell? This was a question that remained unanswered until recently. We know now that, in the cytoplasm, LPS are sensed by caspase-11 in mice and caspase-4 and -5 in humans. In this section, we discuss and review the seminal papers that led to the discovery of caspase-11 as a sensor of intracellular LPS (Figure 4).

Caspases are a family of conserved endoproteases with cysteine protease activity that specifically cleave the peptide bond after aspartic acid residue in the substrate and play central roles in driving apoptosis and inflammation. Inflammatory procaspases (caspase-1, -4, -5, -12 in humans and caspase-1, -11, and -12 in mice) [89,94] require catalytic activation through the stimulated PRRs and subsequent formation of inflammasomes [95,96]. Upon activation, inflammatory caspases process and produce a mature form of inflammatory cytokines (IL-1β and IL-18) and induce pyroptotic cell death [94,97]. In contrast to previous studies that speculated caspase-1 as the sole mediator of pyroptotic cell death, recent studies have revealed a dominant role of caspase-11 in induction of pyroptosis [98,99,100]. Caspase-11 in mice corresponds to caspase-4 and caspase-5 in humans and induces activation of non-canonical inflammasome.

Over a decade ago, caspase-1 and caspase-11 were thought to contribute similar signaling pathways in inflammation due to the fact of their highly homologous sequences [101,102]. Further supporting this hypothesis, in vivo studies with caspase-1 (*Casp1*-/-) and caspase-11 (*Casp11*-/-) knockout mice demonstrated that both were resistant to endotoxin shock induced by LPS when compared to wild-type mice [103,104]. However, in 2011, Kayagaki et al. [104] discovered that the original *Casp1*-/- mice generated using 129 mouse strain embryonic cells were actually deficient in both caspase-1 and caspase-11. Sequencing of 129S1 genomic DNA revealed a 5 bp deletion within caspase-11 locus, generating highly unstable caspase-11 transcripts and, thus, mice on a 129 background essentially have no functional caspase-11. Because of the close proximity of the caspase-1 and caspase-11 loci (~1500 bp) on the chromosome, it is nearly impossible to segregate these genes by recombination; hence, the resultant *Casp1*-/- mice generated on a 129 background are doubly deficient for caspase-1 and caspase-11 [104,105]. Indeed, these *Casp1*-/- mice are now commonly used in research as *Casp1*-/-*Casp11*-/- DKO mice. This discovery necessitated a re-evaluation of the studies that utilized the original *Casp1*-/- mice. To tease apart the functional contributions of caspase-1 versus caspase-11 during LPS-induced endotoxemia, Kayagaki et al.microinjected a caspase-11 bacterial artificial chromosome (BAC) transgene into the *Casp1*-/-*Casp11*-/- DKO embryo to generate mice lacking just caspase-1 (*Casp1*-/-*Casp11*Tg mice) [104]. Interestingly, they found that WT and *Casp1*-/-*Casp11*Tg mice were equally susceptible to LPS shock while *Casp11*-/- mice were resistant. Importantly, during CTB+LPS stimulation or bacterial infection with *E. coli*, *C. rodentium*, and *V. cholerae*, caspase-11 directly promoted pyroptotic cell death that was not dependent on caspase-1. Thus, a search for upstream sensors and regulators of these pathways began. To this end, multiple subsequent studies showed that the TLR4–TRIF–IRF3 signaling axis was critical for activation of the caspase-11 pathway and induction of proptosis [15,106,107].

Furthermore, TLR/IFNAR signaling-mediated complement pathway activation (Cbp1-C3-C3aR) was shown to be required for priming and activation of caspase-11 [108]. However, the first breakthrough came from two independent studies showing that intracellular LPS activated caspase-11 and subsequent pyroptotic cell death [14,109]. Given that CTB promoted intracellular delivery of virulence factors in the host cell cytoplasm, Kayagaki and colleagues hypothesized that delivery of LPS into the cytoplasm of the cell drove non-canonical inflammasome activation and cell death independently of TLR4 [104]. Indeed, transfection of LPS or lipid A (active portion of LPS) was sufficient to promote caspase-11-dependent IL-1β production and cell death in BMDMs [110]. Importantly, LPS or lipid A transfection-induced IL-1β and cell death were still observed in TLR4-deficient BMDMs, suggesting a TLR4-independent recognition of cytoplasmic LPS [110]. In line with these observations, Hagar et al. independently showed that transfection of Gram-negative bacterial lysates or LPS induced cell death that was dependent on caspase-11 [14]. This group demonstrated that specific lipid A structures were required to activate caspase-11; penta- and hexa-acylated lipid A activated caspase-11, whereas tetra-acylated lipid A was not detected, i.e., LPS from *Francisella novicidia* and *Yersinia pestis* that comprise the tetra-acylated lipid A structure do not activate non-canonical inflammasomes [14,109,110], and many pathogens seem to exploit this feature to escape caspase -11 detection [14,111]. In addition to in vitro studies, LPS-induced shock in TLR3-primed mice was demonstrated to be driven by caspase-11 independently of TLR-4, suggesting the presence of a TLR-4 independent LPS sensing mechanism [110]. Altogether, these results clearly demonstrate a novel cytoplasmic sensor that activates caspase-11 and induces subsequent non-canonical NLRP3 inflammasome activation and pyroptotic cell death.

Bringing the search for the elusive cytoplasmic LPS sensor to an end, an elegant series of studies by Shi et al. showed that caspase-4 in humans and caspase-11 in mice directly sense LPS [98]. This was a ground-breaking study given that it identified caspase-4/caspase-11 (inflammatory caspases) as novel cytoplasmic LPS sensors. The discovery of caspase-4/11 as a potential direct LPS sensor was fueled by a serendipitous observation while attempting to generate recombinant caspase-4/11 for biochemical studies. Recombinant caspase-4/11 purified from Sf21 insect cells eluted at a size of ~100 kDa while recombinant caspase-4/11 was eluted from *E. coli* at a size of ~600 kDa. Analytical ultracentrifugation and static light scattering of the ~100 kDa elute showed that recombinant caspase-4/11 from Sf21 were monomers and recombinant caspase-4/11 from *E. coli* were oligomers. These observations suggested that some factors present in the bacteria were causing caspase-4/11 oligomerization. Given that LPS is the most abundant component in *E. coli*, Shi et al. [98] hypothesized that caspase-4/11 may directly bind to LPS, resulting in its oligomerization. Indeed, biotinylated LPS or lipid A efficiently precipitated caspase-4 and caspase-11 in transfected HEK293T or immortalized BMDM cells. These results were further corroborated by surface plasmon resonance experiments where both LPS and lipid A showed a strong dose-dependent association with caspase-4 and caspase-11. Further biochemical studies demonstrated that the CARD domain of caspase-11 was sufficient to bind to LPS. This series of studies demonstrated that the CARD domain of caspase-4/11 directly binds to the lipid A portion of LPS which results in caspase-4/11 oligomerization and subsequent activation of NLRP3 inflammasome and pyroptotic cell death.

While the molecular mechanisms leading to LPS sensing by caspase-11 and subsequent inflammatory processes have been well-established [112,113,114], how LPS makes its way into cytosol remained unknown until recently. High mobility group box 1 (HMGB1), when added with extracellular LPS onto peritoneal macrophages or human THP1 monocytes, induced significant pyroptotic cell death that was dependent on caspase-11 and caspase-4, respectively [115]. High mobility group box 1 directly bound LPS as shown by surface plasmon resonance and pull-down experiments. The HMGB1 A box motif binds to the LPS polysaccharide and B box motif with lipid A, and both motifs are important for HMGB1′s interaction with LPS [115,116]. Given that the requirement for HMGB1 can be bypassed by LPS delivery via cholera toxin B or lipofectamine, Deng and coworkers hypothesized that HMGB1 plays a role in delivering LPS into the cytoplasm [115]. Indeed, they reported that LPS was detected in the cytosol of macrophages only when stimulated with LPS+HMGB1 but not LPS alone. Furthermore, proximity ligation assay experiments showed that the intracellular LPS interacted with caspase-11 in macrophages treated with LPS+HMGB1 but not LPS alone. Because HMGB1 internalization into cells is mediated by its receptor RAGE [117], genetic deletion of RAGE or RAGE inhibition by blocking antibodies both impaired HMGB1-mediated delivery of LPS into the cytoplasm of macrophages [115]. In vivo, TLR4 stimulation of liver hepatocytes promotes HMGB1 production that can then bind extracellular LPS to be delivered into innate immune cells via RAGE.

In addition to HMGB1, a recent study implicated secretoglobin 3A2 (SCGB3A2), a protein primarily secreted by airway cells, in promoting LPS delivery into macrophages and tumor cells to induce pyroptotic cell death [118]. Biotinylated LPS interacted with recombinant SCGB3A2 as demonstrated by pull-down assays. The SCGB3A2 binding resulted in de-aggregation of LPS micelles as demonstrated by crude LPS migration in gels and dynamic light scattering in the presence or absence of SCGB3A2. The SCGB3A2 required syndecan-1 to be present on the cell surface for mobilization of LPS into the cytoplasm. These studies clearly highlight how different host-derived proteins play important roles in binding LPS and delivering them into the cytoplasm. Vanaja et al. also showed that bacterial outer membrane vesicles (OMVs) containing LPS are endocytosed, resulting in subsequent release of LPS into the cytoplasm [119]. Studies have shown that LPS, once inside cells (presumably in endosome/lysosome vacuoles), requires an additional set of regulatory molecules to be released into the cell cytoplasm to be detected by caspase-11. The family of guanylate binding proteins (GBPs) has been implicated in this process of LPS release from the intracellular vacuoles to the cytoplasm. During an active bacterial infection, GBPs and IRGB10 are recruited to the pathogen-containing vacuole and induce lysis of the vacuole to release pathogen products including LPS to the cytoplasm [112,120,121,122,123]. Specifically, one study showed that caspase-11-dependent cell death induced by LPS transfection or LPS+CTB treatment was attenuated in *Gbp*chr3-/- (deficient in GBP on chromosome 3 which includes GPB1, GBP2, GBP3, GBP5, and GBP7) macrophages [123]. Interestingly, the reduction of cell death in the absence of GBPs was only partial, suggesting that GBPchr3-independent mechanisms are also involved.

Altogether, we now have a better understanding of the molecular mechanisms involving recognition of cytoplasmic sensing by caspase-11 that result in pyroptotic cell death. The mechanistic pathways that promote cell death downstream of caspase-11 has been reviewed in detail elsewhere [114]. We are hopeful that future studies will elucidate the specific pathways and redundancies in the shuttling and release of LPS from the extracellular environment to the endosomes and from the endosomes to the cytoplasm. Future studies aimed at understanding how different bacterial pathogens utilize HMGB1 and/or SCGB3A2 and whether the nature of the LPS/caspase-11 interaction changes depending on the pathogens involved will also reveal novel regulatory molecules.

## 6. Summary

The last decade has seen unprecedented progress in our understanding of host LPS recognition systems. From TLR4 as the sole LPS sensing mechanism, we now have two additional LPS recognition systems: the TRP channels and caspase-11. As discussed in this review, all of these recognition systems have non-redundant functions and allow the host different ways and opportunities to swiftly recognize, respond, and clear the LPS-bearing pathogen which is how the host comes into contact with LPS most of the time. Conversely, hyperactivation of these pathways, as is the case in sepsis, often results in multiorgan failure and death of the host without medical intervention. The fact that we have employed so many different ways to recognize LPS underscores the importance of this molecule as a pathogen-associated molecular pattern. These new pathways that have been discovered not only expand our knowledge of the complex LPS sensing pathways but will also pave the way for translational research for the benefit of human health.
